# Creating User Personas to Represent the Needs of Dementia Caregivers Who Support Medication Management at Home: Persona Development and Qualitative Study

**DOI:** 10.2196/63944

**Published:** 2025-07-25

**Authors:** Anna Jolliff, Priya Loganathar, Richard J Holden, Anna Linden, Himalaya Patel, Jessica R Lee, Aaron Ganci, Noll Campbell, Malaz Boustani, Nicole E Werner

**Affiliations:** 1 Center for Research and Innovation in Systems Safety Department of Anesthesiology Vanderbilt University Medical Center Nashville, TN United States; 2 Department of Industrial & Systems Engineering University of Wisconsin - Madison Madison, WI United States; 3 Department of Health & Wellness Design School of Public Health Indiana University Bloomington Bloomington, IN United States; 4 Health Systems Research Center for Health Information and Communication Richard L Roudebush Veterans Affairs Medical Center United States Department of Veterans Affairs Indianapolis, IN United States; 5 Department of Human Systems Engineering Ira A. Fulton Schools of Engineering Arizona State University Mesa, AZ United States; 6 Herron School of Art + Design Indiana University Indianapolis Indiana University Indianapolis Indianapolis, IN United States; 7 Department of Pharmacy Practice Purdue University West Lafayette, IN United States; 8 Indiana University Center for Aging Research Regenstrief Institute Indianapolis, IN United States; 9 Center for Health Innovation and Implementation Science Indiana University Indianapolis, IN United States; 10 Department of Medicine Indiana University Indiana University School of Medicine Indianapolis, IN United States

**Keywords:** medication adherence, user-centered design, digital health technology, informal caregivers, dementia, artificial intelligence, AI

## Abstract

**Background:**

Caregiver-assisted medication management plays a critical role in promoting medication adherence and quality of life for people living with Alzheimer disease or related dementias (ADRD). The current landscape of digital and nondigital interventions to support medication management does not meet caregivers’ needs, contexts, and levels of technological proficiency. Intervention development can be facilitated using personas or data-driven archetypes that represent end users’ traits relevant to solution design.

**Objective:**

This study aims to understand the strategies and unmet needs of ADRD caregivers who manage medications and use this understanding to create personas that can inform customized caregiver interventions.

**Methods:**

Participants were self-identified primary caregivers of people with ADRD living with or near the care recipient. Virtual contextual inquiry was completed in three stages: (1) enrollment interview, (2) virtual observation over a 1-week period, and (3) postobservation interview. Codebook thematic analysis of interview transcripts was used to identify dimensions of caregivers’ approaches to medication management. A reflexive, team-based affinity diagramming approach was used to identify attributes within these dimensions and group attributes into personas.

**Results:**

Participants (N=25) were aged 62.32 (SD 11.86) years on average, and 17 (68%) of them were female. Caregivers varied across 6 dimensions relevant to medication management: strategies for medication acquisition, medication storage and organization, medication administration, monitoring the care recipient for symptoms, communication with care network regarding medication, and acquiring information about medication. Three personas were created to represent the observed strategies, unmet needs, and levels of technology use related to medication management: Checklist Cheryl, in Control; Social Sam, Keeps it Simple; and Responsive Rhonda, Stays Relaxed.

**Conclusions:**

Caregivers in this study demonstrated a range of characteristics and values that informed their approach to medication management. They used a combination of technology-based strategies and strategies situated in their physical environments to manage medications. The personas created can be used to inform interventions, such as digital tools, that address caregivers’ unmet needs.

## Introduction

### Overview

Every year, family and friends of people living with Alzheimer disease or related dementias (ADRD) around the globe provide 82 billion hours of care [1]. These family caregivers assist in promoting the health and safety of the persons living with ADRD, including helping them manage complex medication routines [2-5]. A US-based survey of family caregivers found that medication management is a challenging process due to the considerable time involved, the complexity of routines, fear of making a mistake, and care recipients’ resistance to taking medication [6]. Caregivers of people living with ADRD may face unique challenges; for example, an Australian study of family caregivers found that people living with ADRD may not recognize their need for assistance with managing medication and may feel embarrassed to accept help [7]. International reviews of the literature further suggest that caregivers of people living with ADRD lack training and resources to support the breadth of activities associated with medication management [5,8]. Thus, ADRD caregivers would benefit from interventions that are tailored to their needs, contexts, and levels of technological proficiency to facilitate safe and successful medication management [9]. Intervention development can be facilitated using personas or data-driven archetypes that represent end users’ traits relevant to solution design.

### Background

#### Challenges With Caregiver-Assisted Medication Management

Medication management is a complex, multifaceted process [10]. One aspect of caregiver-assisted medication management (henceforth, medication management) is maintaining a constant supply of medications, which is made more complicated by differences in prescription pack sizes, delays in prescription refill, receiving incomplete prescriptions, and return visits to the pharmacy when concerns or errors are detected [10]. Administering medications is complicated by the intricacies of many medication routines, frequent changes to these routines (including the dose, number, and form of medications), and both caregivers’ and care recipients’ forgetfulness to take medications [5,10]. Caregivers are also responsible for making judgments, such as when to withhold, increase, decrease, or discontinue medication, which involves monitoring the care recipient’s symptoms, medication side effects, and whether medications are having their intended effect [10]. Finally, communication with the care team is a key but ambiguous component of medication management; caregivers may struggle to know when to reach out to prescribers or to feel comfortable reaching out and may receive incomplete information about medications from health care providers (eg, side effects to look out for or changes to a prescription) [10,11]. Thus, medication management can represent significant and taxing work for family caregivers.

#### Caregiver-Assisted Medication Management and Well-Being

Medication management has implications for the health and well-being of both the care recipient and caregiver. For the care recipient, international research spanning the United States, Europe, Canada, Japan, and Australia has linked suboptimal medication management with poorer health [12], poorer management of comorbid conditions [13], costly hospitalizations [14], and premature institutional placement [15]. With assistance from caregivers, medication adherence rates among those with ADRD can match those of their peers without cognitive impairment [3]. Among caregivers, performing medical tasks has been associated with positive outcomes, such as gaining new skills and feeling closer to and less worried about the care recipient [6]. However, medication management has also been associated with greater caregiver burden and stress in international research [7,16-18]. In the United States, nearly 20% of family caregivers who perform medical tasks for the care recipient describe fear of making a mistake, 32% report continuously monitoring for something to go wrong, and 40% report feeling “down, depressed, or hopeless” as an outcome of performing these tasks [6]. Therefore, it is critical to understand the unmet needs of caregivers managing medications as the first step in maximizing the positive and mitigating the negative outcomes for care recipients and caregivers alike.

#### Limitations of Existing Medication Management Tools

Many tools already exist to support medication management, from low-tech tools (eg, plastic cups and sandwich bags) to high-tech tools (eg, electronic pillboxes) [9]. Some caregivers’ needs are met by their existing approaches, and, for them, no intervention may be needed [9]. However, other caregivers report that current low-tech tools, such as pillboxes and reminder notes, do not meet their needs [19]. Pillboxes may not accommodate more complex medication routines, and reminder notes may be ignored over time [19]. While digital interventions abound, a recent systematic review of mobile apps available in Google Play or iTunes intended to assist with medication management suggested that these, too, may not meet users’ needs [20]. The reviewed apps were largely low quality and missing desirable features, such as refill reminders and information about adherence [20]. In a US-based study of family caregivers, participants perceived existing digital interventions for medication management as too elaborate or overwhelming [9]. Thus, it is evident that current interventions to support medication management do not meet the full range of needs, contexts, and interests in technology of the current population of ADRD caregivers. By examining the strategies currently used by caregivers to manage medication, we can better understand the features of interventions that would support medication management [21].

#### A Call for Customized Solutions

Each caregiver’s medication management needs and contexts are different [22]. Needs may vary based on the nature of the routine [23-25], qualities of the care recipient [6,8,26,27], and caregiver characteristics [28,29]. The routine itself is shaped by the variety of medications prescribed, the number of prescribers, and the use of multiple pharmacies [23-25]. Qualities of the care recipient include the stage and symptoms of dementia [8] and any co-occurring medical conditions [6], both of which may change over time, in turn, prompting changes to their medication routine [26,27]. Caregivers vary in their health literacy, access to information, commitment to self-care, and valuing of the care recipient’s independence [28], all of which may affect how they manage medication. Caregivers also differ on the degree to which they receive support from others with caregiving, including medication management [8]. One analysis that identified typologies of caregiver support networks found that male caregivers and low-income caregivers in the United States were more likely to be in low support caregiving networks, that is, networks in which caregivers receive less support for themselves (eg, emotional support and support with housework) or for the care recipient (eg, personal care and transportation) [29]. In summary, the intricacies of medication management intersect with the characteristics of caregivers and care recipients to yield distinct requirements of supportive interventions.

#### Translating Unique Needs Into Solution Design

There is an outstanding need to understand the variable needs of caregivers managing medication on behalf of people living with ADRD and design solutions to meet those needs. Furthermore, in the design of solutions to address these caregiving challenges, it is critical to involve caregivers themselves [30]. User-centered design (UCD) is an internationally recognized approach for integrating future end users into the design of interventions that aim to meet their unique and complex needs [31]. UCD is defined as “an approach to interactive systems development that aims to make systems usable and useful by focusing on the users, their needs and requirements” [31]. UCD engages representatives of the user population in the design of interventions, resulting in interventions that are usable, useful, relevant, and adaptable to caregivers’ needs [21,32]. UCD has proven valuable to the design of solutions in the health care domain across the globe to meet diverse patient population needs [30,33-35].

Within the field of UCD, persona design is a specific method in which real participant data are analyzed and distilled into archetypal end-user representations or personas [36]. Personas can be used to represent the range of key user dimensions regarding a certain process (eg, medication management), including relevant demographic characteristics, strategies they use to do their work, and any unmet needs they encounter [37]. In the context of intervention development, personas have certain advantages over other products of qualitative inquiry, such as theory, themes, or concept maps; they offer concise, data-driven representations of end users, allowing designers to ask and answer the question, *What do our end users need?* [36]. Personas may be particularly helpful for informing digital caregiving designs, including home monitoring systems, automated support with caregiving tasks, social robots that provide comfort to care recipients, and social networks that provide support to caregivers [38]. Persona development is a life cycle; initial personas are somewhat crudely defined by their distinguishing features (conception), enriched with storytelling and stakeholder feedback (gestation), and iteratively refined as they are applied (adulthood) [36]. Previous studies in the United States [39,40], Singapore [41], and Germany [42] have used personas to depict important variations among the target populations of interventions and to infer the necessary intervention features to meet users’ unique needs.

### Study Purpose

This study aimed to answer the following research questions (RQs):

- RQ1—What are the strategies used by caregivers of people living with ADRD who manage medication?
- RQ2—What are the unmet needs of caregivers of people living with ADRD related to medication management?
- RQ3—How can the answers to RQs 1 and 2 be represented as personas to inform intervention development?

## Methods

### Ethical Considerations

This study was subject to an expedited ethics review and received approval from the University of Wisconsin–Madison Institutional Review Board (2021-0339). Participants provided verbal consent to participate and were told that they could withdraw consent at any time. All data were deidentified before analysis. Participants received US $25 for completing the enrollment interview and another US $25 for completing the postobservation interview.

### Study Design

To identify the approaches and unmet needs related to the daily work of medication management for caregivers of people living with ADRD, we adapted a design research method, contextual inquiry, to a virtual form, which we have termed virtual contextual inquiry (VCI) [43,44]. Traditional contextual inquiry combines principles of ethnography with cognitive task analysis, seeking to understand how tasks are completed in naturalistic environments, including the implicit and tacit knowledge obtained from repeatedly performing contextualized work [45]. In traditional contextual inquiry, researchers observe people in their natural environments while they perform tasks of interest (eg, medication management) as they normally would. Interview probes are inserted unobtrusively before, during, and after observation. In VCI, participants send the research team multimedia messages (eg, photos and textual descriptions of work) during or soon after completing the work [43]. These multimedia messages allow for nearly real-time observation of tasks and serve as a cognitive aid during the postobservation interview [46,47]. As with in-person contextual inquiry, in VCI, data collection is structured across 3 phases: before, during, and after observation.

In this study, VCI was selected over traditional contextual inquiry for myriad reasons. First, medication management takes place in various physical spaces and may vary depending on the day [10]. In order to gain a more accurate picture of the work, observation must be close and sustained. However, to observe participants in person over multiple days would be costly and time consuming for the researcher as well as inconvenient and even uncomfortable for participants [48]. Virtual observation thus strikes a balance between practicality and comprehensiveness [46,49]. Virtual observation may also improve the accuracy of data; participants who are less conscious of being observed may be less likely to alter their typical behaviors [50,51]. Furthermore, virtual observation inherently creates artifacts that can be repeatedly referenced throughout analysis, meaning researchers are less dependent upon memory or written notes. Finally, VCI allows for widespread recruitment, rather than recruiting solely from areas within geographic proximity to the research institution, and permits scholarship to continue in the face of circumstances that prevent in-person observation (eg, the COVID-19 pandemic) [48].

### Conceptual Framework

Our conceptual framework for medication management, which influenced the enrollment and postobservation interview process, was based on the findings of Smith et al [10] related to contexts in which medication-related problems can occur for family caregivers managing medications. We were also guided by previous research on crafting personas for intervention design and thus inquired about participants’ unmet needs and technology use [36,52].

### Study Sample

This study used 2 different methods to enroll a convenience sample of caregivers of people living with ADRD. In the first method, participants were recruited from the Wisconsin Alzheimer’s Disease Research Center’s registry of self-identified primary caregivers and in the second method from the research team’s ADRD caregiver registry. The eligibility criteria included (1) self-identifying as a primary caregiver for a person living with ADRD; (2) aged ≥18 years; (3) managing medication for a person living with ADRD, as defined by our conceptual framework; (4) currently living with or close to a person living with ADRD; (5) having access to a cellphone during the study period; and (6) speaking English.

### Study Procedure

#### Recruitment

Participants who were recruited from the Wisconsin Alzheimer’s Disease Research Center emailed the study team if they were interested in participating. The study information sheet was then shared with the participant, and a phone screening was scheduled. Members of the study team’s caregiver registry were first contacted by phone, as they had previously indicated a willingness to be contacted regarding research opportunities. For both recruitment methods, after the screening call was complete and participants provided verbal consent to participate, the enrollment interview was scheduled. Those eligible and willing to participate completed the VCI process, which consisted of three stages: (1) enrollment interview, (2) virtual observation over a 1-week period, and (3) postobservation interview. The interviewer (PL) and participants were unknown to each other before recruitment, and participants knew only the interviewer’s professional status as a doctoral student.

#### Enrollment Interview

The enrollment interview was a 1-hour audio-recorded session that took place over a secure videoconferencing software. During the interview, participants were introduced to the text messaging procedure to be performed during the observation period. Participants were provided with examples of multimedia text messages that would meet study criteria, invited to add the study team’s phone number as a contact in their phone, and practiced submitting a message to the study phone. Participants were also provided with an instructional video that detailed how to submit a message. The interviewer (PL) cautioned participants against submitting messages that contained identifying information. Following this, participants indicated 2 times in the morning and evening at which they would like to receive prompts to submit a message from the study team. The interviewer scheduled a final interview that would occur after the observation period and approximately 8 to 10 days from the enrollment interview. Finally, participants completed a demographic survey and were asked to describe their daily, weekly, and monthly medication management activities. On the basis of this information, the interviewer created an initial list of potential persona dimensions (eg, *medication administration*), which were brought to the research team for discussion and refinement.

#### Observation Period

During the observation period, participants sent multimedia text messages (eg, textual descriptions, still photos, videos, and audio files) that described their medication management to a secure phone dedicated exclusively to study-related communication. Participants were asked to send at least 2 multimedia text messages daily for 7 days, capturing the medication management activities they performed. At the beginning and end of each day, participants received a text message reminding them to send messages. All messages were deidentified to protect the identity of the participants and uploaded to a shared and secure cloud-based drive. After each participant’s observation period, the research team met to review their observation data and made notes of any multimedia messages that needed more clarification in the postobservation interview (eg, if the tasks pictured were not clear or if any essential context was missing). In this meeting, the research team also used the caregivers’ messages to summarize their approach to medication management, which would later be presented to the caregiver for feedback in the postobservation interview.

#### Postobservation Interview

We conducted a 1-hour postobservation interview via videoconferencing software within 8 to 10 days after the enrollment visit. During the interview, an interviewer trained in contextual inquiry reviewed the participant’s multimedia messages and probed for detail. The interview followed the standard procedure for contextual inquiry in that questions did not follow a predetermined interview guide but instead were specific to the data supplied by participants [53]. For example, one participant (P15) shared an image of the spreadsheet they used to track medications. The interviewer asked, “Can you talk a little bit about how you started using that [spreadsheet] or how you came up with that?” Another caregiver (P33) shared over text that the care recipient had missed a dose of their medication, which meant that the caregiver had to remind them to take it. The interviewer asked, “How did you know he missed his medication, and then how did you go about reminding him?” Finally, the interviewer presented the participant with their summarized approach to medication management for reflection and refinement. The interview was audio recorded and transcribed for analysis.

### Analysis

#### Overview

There is no single, standardized approach to persona development [39-42,53,54]. Our approach combined positivist and nonpositivist approaches [55]. We first used a structured, positivist approach to identify dimensions of medication management. We used this approach under the assumption that there were a finite number of medication management activities in which caregivers could engage, many of which could have been anticipated before analyses. It was reasonable to assume that 2 coders could identify the same activities, regardless of their perspectives.

With that in mind, we first used Microsoft Excel to conduct a codebook thematic analysis of postobservation interview transcripts to identify dimensions of medication management [55]. Initial dimensions were those generated by the interviewer in the enrollment interview. Four coders (PL, AL, JRL, and Mengwei Tang) coded the first 2 postobservation interview transcripts as a team. During that process, the coders refined the initial codebook, adding or modifying dimensions, definitions, and examples. The initial codebook was then brought to the entire research team and refined through consensus-based discussion. The codebook was then applied separately to the remaining transcripts by the 4 coders, such that each transcript was coded by 2 coders. To promote reliability, coders met weekly to discuss their coding and resolve any discrepancies through consensus-based discussion [56]. The coding team also met regularly with the full research team to discuss coding and resolve any discrepancies [56]. Any emergent codes or proposed changes to the codebook were brought to the entire research team for discussion and approval. The resulting final codebook represented an enumeration of the initial persona dimensions.

We then adopted a nonpositivist approach to move from dimensions to interpretive stories, in this case, toward personas and their attributes. This shift was consistent with a philosophical assumption held by the research team that, while it is possible to identify objective and discrete dimensions of medication management (eg, *medication acquisition*), we were unlikely to identify discrete types of people through our analysis. The enumeration of attributes within these dimensions and the assignment of attributes to personas were assumed to be subjective and interpretive, a product of the minds that performed the work [55].

To move from dimensions to attributes, we identified passages coded to a specific dimension and created a sentence-length paraphrase of each passage. These paraphrases were transferred to virtual sticky notes, color-coded by participant ID, and grouped by perceived similarity in an iterative team-based affinity diagramming process. For example, within the dimension *approach to medication acquisition*, sticky notes perceived as describing a proactive approach to medication acquisition were grouped together to form an attribute. If it was perceived that participants with this proactive approach to medication acquisition tended to use a strategic approach to storing medications, these attributes were spatially positioned near each other on the virtual sticky board. Each participant was assigned a distinct color sticky note. If, for example, three distinct colors appeared in the same regions of the whiteboard, it practically meant that those three participants shared an attribute. When multiple participants seemed to share multiple attributes, it warranted the creation of a persona. A process of crystallization was used to add depth and nuance to the final personas [57]. Transcripts were critically reviewed by a research team member (PL) to look for information that could add detail to the developed personas, ensure all perceived attributes had been represented by a persona, and check that no persona too closely resembled any one participant. The cowriting of this manuscript allowed the multidisciplinary research team to compare understandings of how attributes fit or did not fit within each persona. Member reflections (a Big Q alternative to member checking) were also used to refine personas [58]. The research team shared drafts of personas with 7 ADRD caregivers who were not involved in the study and augmented the personas based on their feedback. This approach was not intended to *verify* personas but rather add further depth and nuance to the research team’s interpretations.

#### Research Team Positioning

Members of the research team held professional positions that inevitably influenced their perceptions of attributes and the creation of personas. Four members of the research team were faculty (RJH and NEW) or doctoral students (PL and AL) in the field of human factors engineering, which meant that attributes likely to shape or pose a barrier to work performance were naturally emphasized, along with attributes with implications for product design. The lead author of this paper (AJ) was a licensed mental health counselor with training in theories of personality and thus brought assumptions about the likelihood that certain attributes would or would not co-occur. The collaboration of a pharmacist (NC), geriatrician (MB), and a research health scientist specializing in human-computer interaction (HP) added depth of understanding to topics relevant to the RQ.

## Results

### Overview

We reached out to 40 caregivers, and 25 (62%) of them enrolled in the study.

Caregivers who chose not to enroll largely did not respond to our outreach; of those who did respond, 10% (4/40) were ineligible, 3% (1/40) were unable to find time to schedule the enrollment interview, and 3% (1/40) were not interested in sending multimedia messages.

On average, participants were aged 62.3 (SD 11.9) years; 68% (17/25) of the participants were female, and all (25/25, 100%) identified as White and not of Hispanic or Latino origin. The care recipients, on average, were aged 76.7 (SD 11.1) years, and 12% (3/25) were aged <65 years. Among care recipients, 48% (12/25) were female, 92% (23/25) were White, 4% (1/25) were Black or African American, and 4% (1/25) were of Hispanic or Latino origin. All (25/25, 100%) participants demonstrated multiple medication management activities over the course of the 7-day observation period, and 96% (24/25) of the participants submitted messages on at least 5 of the 7 days. Participant characteristics are summarized in Table 1.

**Table 1:** Participant demographics (N=25).

Characteristics			Values
**Sex, n (%)**			
	Female	17 (68)	
	Male	8 (32)	
Age (y), mean (SD)			62.3 (11.9)
**Race, n (%)**			
	Black or African American	0 (0)	
	White	25 (100)	
**Ethnicity,** **n (%)**			
	Hispanic or Latino	0 (0)	
	Non-Hispanic or Latino	25 (100)	
**Highest educational attainment,** **n (%)**			
	High school diploma or equivalent	1 (4)	
	Technical school, vocational training, or community college	5 (20)	
	4-year college	8 (32)	
	Postcollege education	11 (44)	
**Employment status,** **n (%)**			
	Full time	4 (16)	
	Part time	3 (12)	
	Not working	4 (16)	
	Retired	14 (56)	
**Annual income (US $),** **n (%)**			
	<20,000	1 (4)	
	20,000-39,999	3 (12)	
	40,000-$59,999	5 (20)	
	60,000-79,999	6 (24)	
	80,000-99,999	5 (20)	
	>100,000	2 (8)	
	Undisclosed	3 (12)	
Length of time managing medications for care recipient (y), mean (SD)			5.0 (3.1)
**State of residence,** **n (%)**			
	Wisconsin	16 (64)	
	California	5 (20)	
	Other	3 (12)	
	Undisclosed	1 (4)	
**Care partner’s relationship to the care recipient,** **n (%)**			
	Adult child or child-in-law	8 (32)	
	Spouse or significant other	16 (64)	
	Other (mother and father)	1 (4)	
**Care recipient’s living arrangement,** **n (%)**			
	Lives in the primary caregiver’s home	20 (80)	
	Lives in the care recipient’s own home	4 (16)	
	Other (independent living facility)	1 (4)	

**Figure 1:**
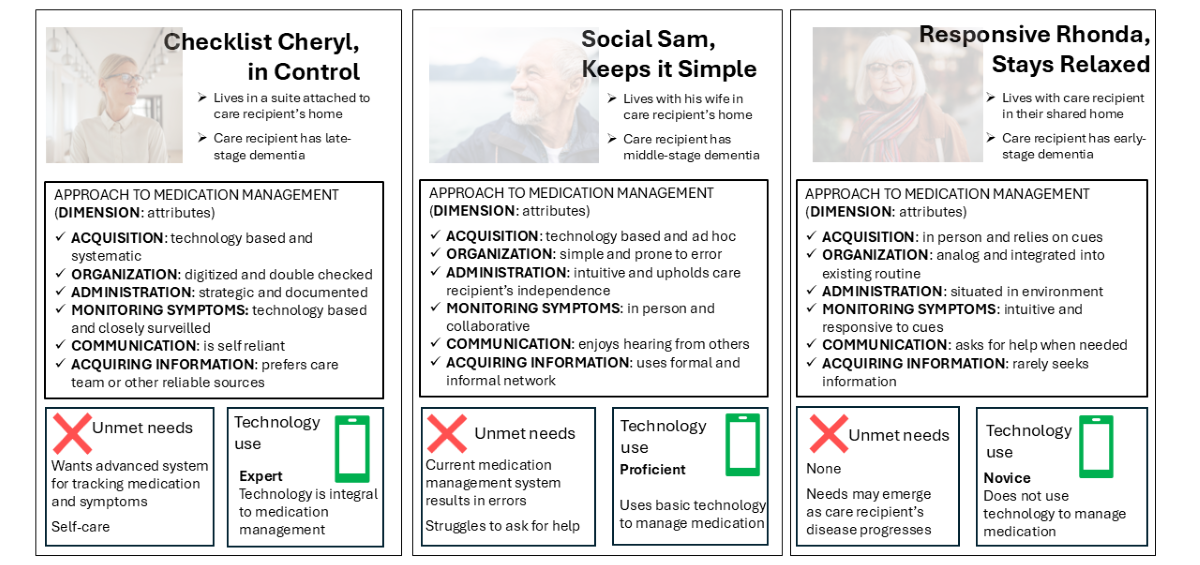
Three persona profiles representing caregivers' medication management for patients with Alzheimer disease or related dementias.

We derived 3 personas: Checklist Cheryl, in Control; Social Sam, Keeps it Simple; and Responsive Rhonda, Stays Relaxed. These personas varied across 6 dimensions relevant to medication management: approach to medication acquisition and organization, medication administration, monitoring the care recipient for symptoms, communication with the care network regarding medication, and acquiring information about medication. The presentation of the personas given subsequently is organized by their approach to each dimension of medication management, with illustrative examples and quotations from real study participants. Persona profiles are presented in Figure 1.

### Checklist Cheryl, in Control

#### Overview

Checklist Cheryl, in Control, is characterized by her desire to do things on her own, be in control, and create reliable and precise systems and regimens. Cheryl lives in a suite that is separate from but connected to the home of the care recipient, who has late-stage dementia.

#### Approach to Acquiring and Organizing Medication

Checklist Cheryl has an organized, technology-based system for acquiring and maintaining a constant supply of medications. She sets up automated refills and mail-based delivery for all medications. She organizes medication in an automated medication dispenser, which she fills every 2 weeks. When filling the dispenser, she verifies the dosage in a notebook that describes each medication as well as a large, printed list of the medications with pictures and a description of each that she keeps on the wall for her reference.

#### Approach to Administering Medication

Checklist Cheryl continuously improved upon her strategies for medication administration and documents the effectiveness of these strategies in a journal. One participant described iterative strategizing to encourage the care recipient to take her medication:

[The care recipient] didn’t like [taking medication], and she made bad faces. So, I thought, one day I’ll just give her something extra. It started, I mean, we’ve done like Pringles and different types of chips and crackers and all that kind of stuff.

P1

#### Approach to Monitoring for Symptoms

Checklist Cheryl closely monitors the care recipient for symptoms and writes down her observations in a journal to track changes in behavior. She installs a video surveillance system in the care recipient’s home to better monitor any symptoms or changes in the care recipient’s behavior:

If someone goes in the laundry room, it just brings up my cam and app, and then I can just click on the laundry room, and I can quickly look and see who went in there and what they’re doing.

P1

#### Approach to Acquiring Medication Information

Checklist Cheryl seeks formal advice about medications. She contacts the care recipient’s health care team with medication questions:

The medications he’s getting now are from the neurologist. So if I see something is wrong, I call the neurologist for changes or advice.

P7

Alternatively, Cheryl may do her own research online. Cheryl does not rely on support groups, friends, or family for medication information.

#### Approach to Communicating With Care Network

Checklist Cheryl was unlikely to ask her formal care team or network of family and friends for help with medication management:

I do all of it. And I order [the medications] myself. Hospice said they would order [the care recipient’s medications], but I order [the care recipient’s medications] at the same time I order my husband’s, so I might as well do it all. I’m kind of a control freak.

P40

#### Technology Use

Checklist Cheryl is a technology expert. Technology is integral to her medication refill system, medication dispenser, information-seeking approach, and the surveillance system she uses to monitor the care recipient’s behavior.

#### Unmet Needs

Checklist Cheryl wants a better system for tracking the care recipient’s changing symptoms and side effects over time. She is also unsure when to communicate symptoms or questions to the formal care team versus relying on the answers she can find through her own research. Finally, she finds that medication management is all consuming and leaves little time for self-care, which often leads to stress.

### Social Sam, Keeps It Simple

#### Overview

Social Sam, Keeps it Simple, is characterized by his attributes of being collaborative, seeking efficiency, and valuing the care recipient’s independence. Sam and his wife live in the home of the care recipient, who has middle-stage dementia.

#### Approach to Acquiring and Organizing Medication

Social Sam has a simple system for monitoring and restocking the medication supply. When he sees a pill bottle running low, he places it near his office computer:

[The medication bottles] will be right in front of me on top of the, my desk where my computer is...I’ll just get on the website and put in the order right then and there.

P8

Sam refills an analog medication organizer on the same evening each week. When possible, Sam incorporates the storage of medication into the care recipient’s existing routine. For example, one participant described storing inhalers in the care recipient’s backpack:

When [the care recipient] used to work, he had that backpack with him 100% of the time, and so that’s where the inhalers were. And that, I don’t want to change that for him.

P10

#### Approach to Administering Medication

Social Sam’s routine for administering medication is so consistent that it has become second nature:

It’s the first thing that I do when I get up...the first thing I do is grab the pill. I also have medication I’m giving the cats, so I grab that at the same time and put it all out on the coffee table, where I keep it during the day.

P8

Because Sam values the care recipient’s independence, he is subtle when checking whether medication has been taken:

I just pretend I’m doing something else and watch [him take the medication].

P10

#### Approach to Monitoring for Symptoms

Social Sam collaborates with his wife, who is also a member of his care network, to monitor the care recipient for symptoms or changes in behavior:

One of the things that’s happened is [the care recipient] has gotten GERD, probably from all the medication that she’s taking, upsetting her stomach. So she’s belching more...And so [my wife] and I talk all the time...There’s a constant that we check in, how is she? Is that medication bothering her?

P25

To monitor the care recipient’s pain symptoms, Sam takes weekly glances at the as-needed pain medication bottle to see if there are fewer pills than there were the previous week.

#### Approach to Acquiring Medication Information and Communication With the Care Network

Social Sam enjoys receiving information and help from others, both clinicians and nonclinicians. For example, he prefers to pick up medications in person so he can ask the pharmacist questions. Sam uses informal sources of medication information, such as support groups and Facebook:

I try to draw on everyone and try to get the best information possible to make the right decision.

P2

#### Technology Use

Social Sam is technology proficient. He uses text messaging to communicate with his wife regarding medication management and uses phone reminders to prompt himself to do medication-related tasks.

#### Unmet Needs

Social Sam finds his current medication refill reminder system to be too simplistic, not accounting for medications with different refill dates. This insufficiently nuanced system results in Sam occasionally forgetting to refill medications and, consequently, missing doses. Though Sam is naturally collaborative, he finds coordinating medication management activities with other family members and formal care team members to be challenging, as he does not like to bother people.

### Responsive Rhonda, Stays Relaxed

#### Overview

Responsive Rhonda, Stays Relaxed, is characterized by the attributes of being laid-back, nonintrusive, and responding to (rather than preparing for) challenges with medication management as they arise. Rhonda lives with the care recipient, who has early-stage dementia.

#### Approach to Acquiring Medication

Responsive Rhonda responds to overt reminders and alerts to order medication. For example, she may see the medication supply and notice it is low, or she may look into refilling medications when at the pharmacy for other reasons:

Like if I’m going in to pick something up in, at the pharmacy, I will just show them his number, his medical number also and just say, hey, is there anything that’s available for pickup?

P10

#### Approach to Organizing and Administering Medication

Responsive Rhonda relies on nontechnological cues to remind herself to organize and administer medications. She stores pills where she regularly performs her nighttime routine:

The pill is just part of the setup that I do every night, so it’s actually not too hard to remember to put that out.

P9

As part of existing daytime routine of Rhonda, she walks past the medication she set out and notices if the care recipient has taken the medications or if they need to be reminded.

#### Approach to Monitoring for Symptoms

Responsive Rhonda does not have a formal system to monitor the care recipient for symptoms, instead relying on intuition:

And if I feel like, this is more intuitive, is if I feel like he’s [using Tylenol] much more than normal, then that will trigger me to go check on, you know, to ask, dig in a little more about his pain.

P1

Rhonda also relies on the care recipient to report symptoms to her.

#### Approach to Acquiring Medication Information and Communication With the Care Network

Responsive Rhonda is comfortable relying on others in the care network for help, although at this stage in the care recipient’s disease, she rarely needs help. When Rhonda is away, she asks other family members to put the medications out for the care recipient and ensure that they take them. Rhonda seldom sees the need for medication information or advice, but when she does have questions, she is comfortable asking the care recipient’s care team.

#### Technology Use

Responsive Rhonda is a technology novice. Although proficient with mobile phone calls, she does not think of incorporating technology into medication management.

#### Unmet Needs

Responsive Rhonda does not currently perceive any unmet needs related to medication management.

## Discussion

### Principal Findings

In this study, we leveraged the UCD method of VCI to understand ADRD caregivers’ approaches to and needs for medication management. Caregivers exhibited a range of characteristics and values that informed their approach to medication management.

In addition, caregivers used a combination of technology-based strategies and strategies situated in their physical environments to manage medications. However, these personas must be understood with awareness of the relatively homogenous sample, which may limit generalizability. The average caregiver in this study was a retired or unemployed White woman in her 60s, with college or postcollege education, caring for someone in their 70s who lived in her home in the United States. These personas can be used to inform the UCD of interventions for caregivers with similar attributes by enabling designers to build empathy with future users, remain focused on users’ needs, and maintain an empirical foundation for design decisions [36,41,42,54]. Specific implications of designing medication management interventions for the described personas are presented in Table 2.

**Table 2:** Design implications for Checklist Cheryl, Social Sam, and Responsive Rhonda.

Persona	Unmet needs	Example design implications
Checklist Cheryl, In Control	No system for tracking changing symptoms and side effects over time Lacks certainty about when to communicate symptoms and side effects to health care professionals Medication management is all consuming and leaves little time for self-care	Adaptive tools to track data (symptoms, side-effects, and strategies) and display trends AIa-powered database that summarizes academic research Smartphone apps that combine medication management with self-care for caregivers Bridge to state and federal caregiver grants
Social Sam, Keeps it Simple	The current reminder system too simplistic, not accounting for different refill dates Wants more help and advice from family or friends in his care network	Text- or email-based reminder systems for reordering different medications Collaborative platforms for distributing medication management across the care network Social media platforms, virtual support groups, and online forums for obtaining practical and emotional support from caregiver peers
Responsive Rhonda, Stays Relaxed	No unmet needs perceived, but may emerge as the care recipient’s disease progresses	Printed educational materials about ADRDb and its progression Analog tools or infographics that she can situate in her environment A technology broker who can introduce her to digital medication management supports Bridge to local aging and disability resource centers

^a^AI: artificial intelligence.

^b^ADRD: Alzheimer disease and related dementias.

The specific attributes of each persona build on the existing literature and extend knowledge in the domain of medication management. In a US-based sample of older adults with chronic heart failure, Holden et al [40] identified the rule-following persona, who has the tendency to respond to uncertainty by seeking reliable rules and clinical expertise. In this study, Checklist Cheryl demonstrates how this tendency extends to medication management processes; Cheryl painstakingly triple checks her medication organization, performs elaborate documentation, and does not trust family or friends to assist in medication management, meaning much of the workload falls on her. Caregivers such as Checklist Cheryl would greatly benefit from state or federal grant programs that pay for respite care or in-home nursing [59]. Such programs require support from the policy makers who set funding priorities and establish budgets. Programs such as these would allow Cheryl to construct a formal support network that she can trust to offset her personal workload.

There are few digital health interventions to support medication management for caregivers, such as Checklist Cheryl, in Control, with some exceptions found internationally [60-62]. As a technology expert, Cheryl may benefit from a multicomponent smartphone app that allows her to, first, view medications and their doses and verify if they have been administered correctly. Second, such an app could enable Checklist Cheryl to document and visualize trends over time, including trends in the care recipient’s symptoms, medication side effects, and the effectiveness of strategies [63]. Third, because Cheryl is independent and prefers learning from experts over peers, she may benefit from a feature that uses artificial intelligence to answer medication- and dementia-related questions by synthesizing academic literature [64]. Fourth, Checklist Cheryl needs digital assistance with self-care. A recent international systematic review of mobile apps for family caregivers found only 1 app with a self-care feature [65]. Another systematic review of mobile apps with a caregiver and patient focus found that only 15% of apps included a focus on caregiver self-care [66]. Apps that target self-care needs of other types of busy caregivers (eg, new mothers) could be adapted to fit the self-care needs of caregivers of people living with ADRD [67]. While the typical participant in this study was in their early 60s, younger caregivers are more likely to access the internet and use smartphones and thus may be as interested as Cheryl in digital tools for medication management [68]. Digital tools are also likely to have international applications, as over half of the global population owns a smartphone and about one-third of global smartphone owners have used their phone to access health services [69].

Caregivers such as Social Sam, Keeps it Simple, differ from Checklist Cheryl in that they want simple, rather than complex, medication management systems; deemphasize surveillance in favor of promoting the care recipient’s independence; and desire collaboration with other network members as opposed to working alone. While Social Sam’s current system is designed for efficiency, it is not sufficiently sophisticated to accommodate polypharmacy, which is increasingly common as the disease progresses [26,70]. Caregivers such as Sam are less technology proficient than those such as Cheryl; he has incorporated the web version of the pharmacy website into his medication acquisition process, but has not used smartphone apps. This may be consistent with other caregivers of Sam’s age; research affirms that, though only 61% of those who are aged >65 years own a smartphone, 75% of people in this age range use the internet. Technologically proficient caregivers such as Social Sam may benefit from websites that support reordering and administering medications, for example, through text- or email-based reminders [71,72].

Social Sam finds his network of family and friends to be helpful, leveraging them to monitor side effects and make clinical judgments. However, similar to other caregivers, those represented by Sam find it challenging to ask others for help [73]. Similar to other male caregivers, in particular, the support network of Sam is small [29]. The current landscape of digital tools for caregivers that support peer interaction is limited [66]. Social Sam would benefit from web-based tools that support distributing caregiving work, and specifically medication management, across a caregiving team [74,75]. Furthermore, Sam would benefit from interventions (eg, virtual support groups and social media platforms) that enable caregivers to connect with peers beyond their geographic area. In the United States, technologies that connect caregivers may have even greater uptake among members of racial and ethnic minority groups, who use social media such as Instagram, Reddit, Snapchat, WhatsApp, and TikTok at higher rates [76]. Reliance on caregiving support from family and friends is also greater among ethnic minority caregivers [77]. More broadly, technologies that connect support networks are needed in low- and middle-income countries, where reliance on unpaid support for dementia caregiving is more common [78].

Caregivers in this study who are represented by Responsive Rhonda, Stays Relaxed, differ from both Checklist Cheryl and Social Sam in that they adopt a fundamentally laid-back management style with little systemization. These caregivers await and respond to medication issues rather than proactively anticipating and addressing them. Similar to the disengaging persona by Holden et al [40], Rhonda does not actively seek out medication information or experiment to improve processes. Responsive Rhonda may represent those caregivers of people in the early stages of dementia, who tend to take fewer medications than those in the later stages [26,27]. As the care recipient’s disease progresses, Rhonda would likely benefit from caregiver education and training. Those who are caring for someone in the earlier stages of dementia may be relatively disconnected, with healthcare providers as their sole brokers of support; therefore, health care systems can use personas to create tailored discharge instructions for caregivers such as Responsive Rhonda [79].

While she does not currently report unmet needs related to medication management, as the care recipient’s medication regimen becomes more complex, Responsive Rhonda may require a more sophisticated system for medication management. The design of such a system should leverage the tendency of Rhonda to distribute cognition to her physical, rather than digital, environment [80-82]. Rhonda represents caregivers who are novice technology users; therefore, suggestions for Rhonda may also apply to less educated caregivers, who may be less likely and less willing to use digital devices (eg, websites and smartphone apps) to support health, according to a sample of Australian adults [83]. Later in the caregiving journey, caregivers such as Rhonda may benefit from printed materials as well as analog tools (eg, medication organizers) that can be placed as convenient cues throughout the home [80,84]. Rhonda may also benefit from a technology broker who can provide training on using technology to support medication management, for example, by introducing her to specific, informative websites or the mobile app of her preferred pharmacy [85]. This recommendation extends to the 38% of the global population who live in areas in which mobile internet is accessible but who do not currently use it [69].

### Limitations and Future Directions

Our study had limitations. While our average participant mirrored the typical US caregiver in many ways [86] and the average care recipient’s age matched that of many living with ADRD internationally [87], other sociodemographic characteristics were less representative of caregivers. Our participants had, on average, higher educational attainment, which may result in greater health literacy and enhanced medication management as a result [88]. Furthermore, our sample of caregivers was entirely of White individuals. An analysis of Latino caregivers, for example, would have yielded different personas, as Latino caregivers are more likely to be younger, adult children of care recipients and are less likely to live with the care recipient [86]. One US-based systematic review found that Mexican American, African American, and Korean American caregivers are more committed to keeping care recipients at home rather than long-term care facilities, which may result over time in greater caregiver workload [28]. Moreover, our personas reflect only caregivers who have access to and are willing to participate in research projects, and this group may have unique characteristics (eg, connectedness to academic centers and time to participate in research) [40]. Our personas should not be considered representative of all caregivers managing medication but rather a basis on which future investigations may build [89].

These personas are situated between the conception and gestation stages [36]. That is, while our personas reflect important aspects of our data, have been enriched with storytelling, and deepened through stakeholder involvement, they need further refinement before their full implications can be realized. One limitation of these data is that they reflect only 1 week of caregivers’ medication management. A next step in deepening these personas is to collect data about medication management over the course of years, rather than 1 week, to reflect how caregivers’ needs change with disease progression. Future work could also incorporate caregivers of people whose cognitive status had been objectively assessed rather than reported by caregivers. While research suggests that family member reports of dementia are often accurate and, when inaccurate, err on the side of underreporting dementia [90,91], it would be important to understand any differences in medication management associated with objectively confirmed dementia diagnoses. Finally, this analysis focused on process- and strategy-related differences in medication management among caregivers. Personas could be further deepened by analyses focused on cognitive, social, or clinical dimensions of medication management [39].

Both our data collection method and the use of personas carry a risk of forgoing the nuance in participant experiences. Our data consist of messages submitted by caregivers and researchers’ follow-up inquiries regarding these messages. When data are collected virtually and participants are asked to provide explanations in retrospect, some degree of detail may be lost (though this is curtailed using messages to aid recall) [92]. Furthermore, in VCI, participants are unlikely to submit messages in inopportune moments, meaning there is a delay not seen in traditional contextual inquiry between performing the task and reporting upon it [46]. While virtual observation may curtail observer effects, it is also possible that participants curate a more flattering self-presentation when communicating digitally versus in person [22]. Last, persona creation inherently emphasizes points of distinction rather than similarities across participants [40]. It is likely that many real caregivers possess qualities of more than one of the personas identified in this study or resemble different personas at different points in their caregiving journey.

### Conclusions

This study used a novel methodology, VCI, to understand in rich detail how caregivers manage medications on behalf of those living with ADRD. These data were synthesized into user personas that have direct implications for intervention development. For example, smartphone apps that use artificial intelligence to synthesize information about medication management or meaningfully connect caregivers with others who manage medications could support caregivers who value expertise, such as Checklist Cheryl, or collaboration, such as Social Sam. Before the benefit of medication management personas can be fully realized, these personas must be deepened to reflect nuance and expanded to reflect a more diverse group of caregivers and caregiving experiences. These are vital directions for future research. Once personas have matured in this way, they represent a powerful user-centered strategy to test, evaluate, and disseminate technologies that support medication management among ADRD caregivers.

## Abbreviations

ADRD: Alzheimer disease or related dementias
RQ: research question
UCD: user-centered design
VCI: virtual contextual inquiry
